# Measurement of the Thermal Effect of Standing Surface Acoustic Waves in Microchannel by Fluoresence Intensity

**DOI:** 10.3390/mi12080934

**Published:** 2021-08-06

**Authors:** Yiqing Li, Shoupeng Wei, Tengfei Zheng

**Affiliations:** 1 School of Mechatronic Engineering, Xi’an Technological University, Xi’an 710021, China; yi@dr.yitsi.org; 2 School of Mechanical Engineering, Xi’an Jiaotong University, Xi’an 710049, China; s.wei@softdynamics.org

**Keywords:** standing surface acoustic waves (SSAWs), fluorescent intensity ratios, temperature

## Abstract

Temperature is an important parameter for many medical and biological applications. It is key to measuring the temperature of acoustofluidics devices for controlling the device’s temperature. In this paper, Rhodamine B was used to measure the temperature change of the microchannel induced by the SSAWs’ thermal effect in microfluidics. A thermocouple was integrated into the microfluidics device to calibrate the relationship between the fluorescent intensity ratios of Rhodamine B and the temperature. Then, the fluid temperature in the microchannel heated by the SSAWs was measured by the fluorescent signal intensity ratio in the acoustofluidics device. The fluid temperature with different input voltages and different flow rates was measured. The results show that SSAWs can heat the still fluid rapidly to 80 °c, and the flow rates will influence the temperature of the fluid. The results will be useful for precisely controlling the temperature of acoustofluidics devices.

## 1. Introduction

Acoustofluidics, which integrates acoustic waves with microfluidics to manipulate particles and fluids in microscale fluid, has been widely used in material engineering [1,2,3] and biotechnology [4,5], due to the advantages of noncontact, better biocompatibility, and compactness [6]. Surface acoustic waves (SAWs), which are generated and propagated on the surface, have attracted attention for the following reasons. First, SAWs have a high vibration velocity and acceleration and concentrate most of the energy on the surface, resulting in remarkably effective and efficient fluid-structural coupling [7]. Second, SAW devices are easy to fabricate and integrate with microfluidics [8]. Third, SAW devices are inexpensive and simple and can be made portable and flexible. As a result, SAWs have been widely used in acoustofluidics [9].

In the propagation of waves, SAWs will induce acoustic radiation force, acoustic streaming, and a thermal effect in microfluidics [10]. The three physical phenomena interact with each other in acoustofluidics [11]. It is important to study the mechanism of the three physical phenomena for better controlling the particle, droplet, and fluid in acoustofluidics [12]. The mechanisms of acoustic radiation force and acoustic streaming have been deeply studied and widely used in microfluidic systems [13]. The study of SAWs’ thermal effect is still in its infancy. Most researchers have paid attention to the thermal effect induced by travelling surface acoustic waves (TSAWs) in open microfluidics [14]. The droplet thermal effect induced by TSAWs in open microfluidics was observed by the thermal infrared imager and provided a condition for controlling the droplet temperature and heating the micro system [14,15,16,17]. The TSAWs’ thermal effect was also used as energy for thermal drug release from temperature-sensitive liposomes [18], assisting closed-vessel Suzuki coupling reactions [19], lysis cells, and the isothermal amplification of DNA [20,21]. Sung et al. observed the thermal effect of polydimethylsiloxane and developed an acoustothermal tweezer for sorting droplets [22,23,24]. The results demonstrate that the SAWs’ thermal effect plays an important role in microfluidics. It is necessary to measure the thermal effect induced by SAWs.

Standing surface acoustic waves (SSAWs) have been widely used in manipulating particles [25], cells [26], and droplets [27]. Researchers found that the SSAWs’ thermal effect is key in the application of acoustofluidics because of the destruction of bio-particles [28]. To overcome the high temperature in acoustofluidics, cooling equipment had to be integrated into microfluidics [6,29]. However, few have measured the temperature induced by the SSAWs’ thermal effect in the microchannel, which limited the precise controlling and application of particles in the biological detection of SSAW-based devices. In acoustofluidics, there are two difficulties in measuring the temperature in the microchannel. First, the channel size is on the micro-scale, which makes it difficult to integrate contact sensors. Second, the vibration of the substrate and the acoustic streaming will both influence the detection precision of contact sensors. Rhodamine B (RhB) dye, with a high sensitivity to temperature, negligible pressure sensitivity, and independent absorption of PH, has been widely used in the measurement of temperature in microfluidics [30,31,32,33].

In this paper, the fluorescent signal intensity ratios of Rhodamine B were used to measure the temperature changes induced by SSAWs’ thermal effect in microfluidics. The fluorescent signal intensity ratios were calibrated by a thermocouple in the microchannel. Then, the Rhodamine B was used to measure the temperature in acoustofluidics. The temperature of different input voltages and different fluid velocities was measured. The results show that SSAWs can rapidly heat microfluids, and fluid velocity can influence the temperature. This paper will promote the precise control of particles, cells, and droplets in acoustofluidics.

## 2. Experimental Section

### 2.1. Fabrication Process

#### 2.1.1. Fabrication of Calibration Microfluidic

**Figure 1:**
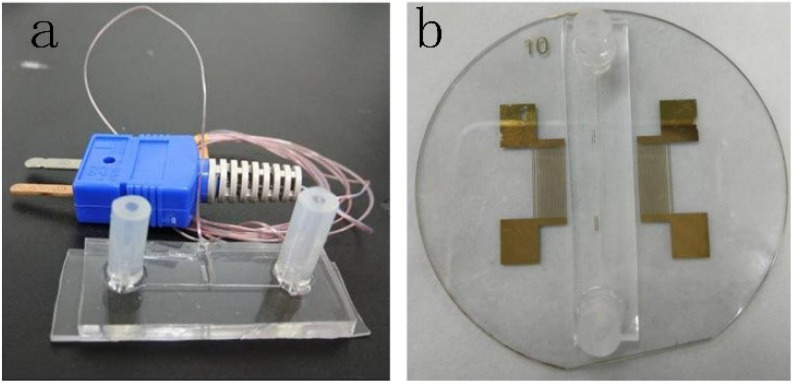
The microfluidic devices. (**a**) Microfluidic device for calibrating the relationship between the fluorescent signal intensity ratios and the temperature. (**b**) Acoustofluidic device for heat RhB solution.

The PDMS microchannel was fabricated by a standard soft-lithography. The mold was made by patterning SU-8 photoresist on silicon. Then, PDMS channel (width: 100μm, height: 100μm) was made by replica process. Two holes of the inlet and outlet were punched with a Harris Uni-core punch in PMDS channel. Then the PDMS channel and silicon were treated by a plasma cleaner with oxygen plasma for 60 s. The PDMS channel and thermocouple (F52-II, Fluke) were aligned and bonded to the silicon (as shown in Figure 1a). The microfluidic was cured at $95{\;}^{\circ }$C for 2 h.

#### 2.1.2. Fabrication of Acoustofluidic

First, the patterning of two interdigital transducers (IDTs) was designed and patterned on the mask. The IDTs comprise 20 pairs of straight fingers with a period of 400μm. Then the patterning was transferred to a 1 mm thick piezoelectric (Lithium niobite, LiNbO3) substrate by the process of lithography. Finally, the SSAW device was fabricated by the process of lift off. The IDTs were made by depositing dual layer metal (Au 200 nm/Cr 50 nm) on the LiNbO3 surface. The PDMS microchannel (width: 100μm, height: 100μm) was fabricated by a standard soft-lithography. The PDMS channel and SSAW device were treated by a plasma cleaner with oxygen plasma for 30 s. The PDMS channel was aligned and bonded to the LiNbO3 surface to form acoustofluidic device (As shown in Figure 1b). The acoustofluidic device was cured 3 h in $150{\;}^{\circ }$C.

### 2.2. Calibration Experiment

The experimental system consists of a water bath kettle (HH.S 11-‘1, Shanghai Boxun), a peristaltic pump (BT100L, Baoding Lead Fluid), and inverted fluorescence microscope (Ti-S Nikon). RhB solution was heated in the water bath kettle. Then, the solution was pump into the inlet of the microchannel through a silicone tube. The thermocouple was used to record the RhB solution temperature. The microchannel was put on the microscope. The microscope recorded the pictures to analyze the temperature of the RhB solution. The temperature measured by thermocouple and RhB was used to calibrate the relationship between the fluorescent signal intensity ratios and the temperature.

The concentration of RhB solution was 0.006 g/L. The flow velocity of the RhB solution was 1000 μL/min. The power of the laser was 100 w when the voltage was 12 V. The environmental temperature was $20{\;}^{\circ }$C.

### 2.3. Temperature Measurement of Acoustofluidic

The experiment platform consisted of two systems. The first system was used to generate SSAWs and heat RhB solution, which consisted of a function generator (Tektronix AFG 3251C, Beaverton, OR, USA), amplifier (NF BA 4850, Kohoku-ku, Japan), and acoustofluidic device. The second system was used to measure the temperature of RhB solution, which consisted of a peristaltic pump and an inverted fluorescence microscope. The acoustofluidic device was put on the microscope. RhB was pumped by the peristaltic into the microchannel. Then the microscope was opened to record the fluorescent signal intensity. An AC signal with the frequency of 15.59 MHz, which was generated by the generator and amplified by the amplifier, was applied on the IDTs. The applied signal simulated the SSAWs to heat the RhB solution. The fluorescent signal intensity of the solution was recorded by the microscope.

## 3. Results and Discussion

### 3.1. The Relationship of the Fluorescent Intensity and Temperature

The relationship between the fluorescent signal intensity *I* and temperature *T* can be expressed by the equation:

(1) $$I\left(T\right)=I_{0}C\varphi \left(T\right)\epsilon$$

where $I_{0}$ is the intensity of the laser, C is the concentration of RhB in the solution, φ(T) is the luminescent intensity of RhB, and ϵ is absorption coefficient. Because of the linear relationship between the luminescent intensity and the temperature, the luminescent intensity can be fitted with a linear approximation in three regions: temperature region of 150 K to 200 K, 240 K to 360 K, and 380 K to 500 K [33]. The luminescent intensity of RhB φ(T) can be expressed as

(2) φ(T)=a+bT

where *a* and *b* are the fitting coefficients.

To eliminate the experimental error, the relationship of relative luminescent intensity *I* and relative temperature difference ΔT is used to measure the fluid temperature,

(3) $$\Delta T=T-T_{0}$$

(4) $$I\left(\Delta T\right)=I\left(T\right)-I\left(T_{0}\right)$$

where $T_{0}$ is the environment temperature, and $I(T_{0})$ is the luminescent intensity of RhB when the temperature is $T_{0}$. Substitute Equations (2)–(4) into Equation (1):

(5) I(ΔT)=c+dΔT

where *c* and *d* are the fitting coefficients. Equation (5) represents the linear relationship between the luminescent intensity and the relative temperature. The linear relationship can be used to measure the temperature by luminescence after the coefficients are made sure.

**Figure 2:**
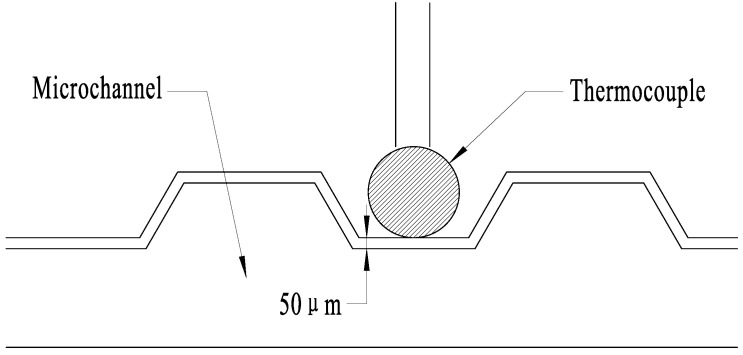
Schematic of the thermocouple relative to the microchannel.

**Figure 3:**
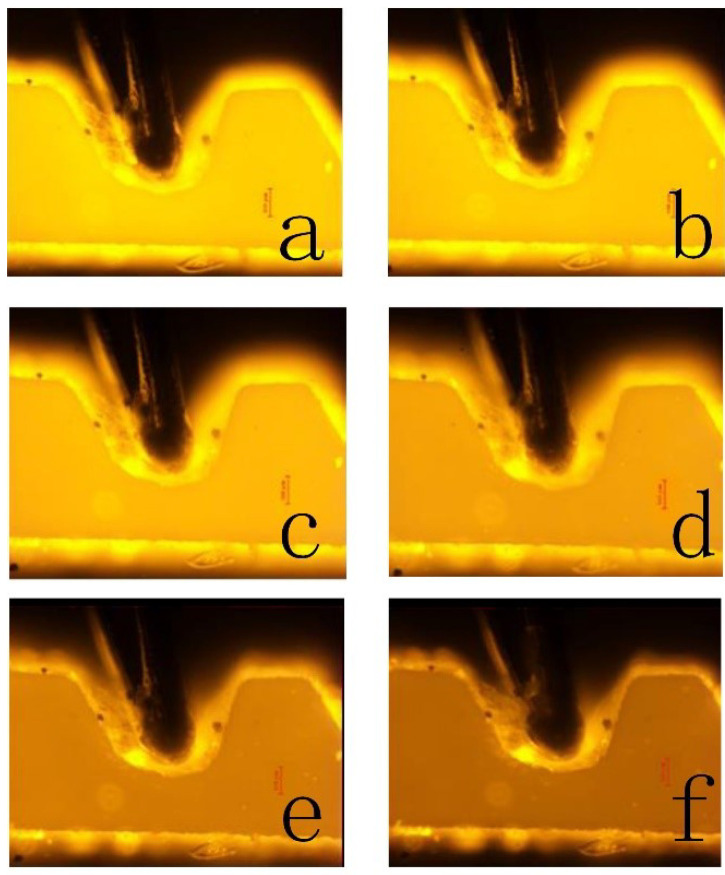
The pictures of RhB luminescent intensity when the temperature is (**a**) 21.8 ${}^{\circ }$C, (**b**) 28.8 ${}^{\circ }$C, (**c**) 34.5 ${}^{\circ }$C, (**d**) 40.8 ${}^{\circ }$C, (**e**) 47.2 ${}^{\circ }$C, and (**f**) 53.4 ${}^{\circ }$C.

To obtain the coefficients, five experiments were performed using the SSAW device. Three pictures were taken at the same temperature in each experiment to obtain the exact temperature. The temperature was measured by the thermocouple that was embedded in the microchannel. Because the distance between the thermocouple and the fluid is less than 50 μm, and the heated solution passes continuously for 5 min, the thermocouple temperature can represent the fluid temperature. Figure 2 shows the schematic of the thermocouple relative to the microchannel, and Figure 3 shows the RhB luminescent intensity of one time. It is obvious that the picture brightness decreases with the increase of the temperature.

**Figure 4:**
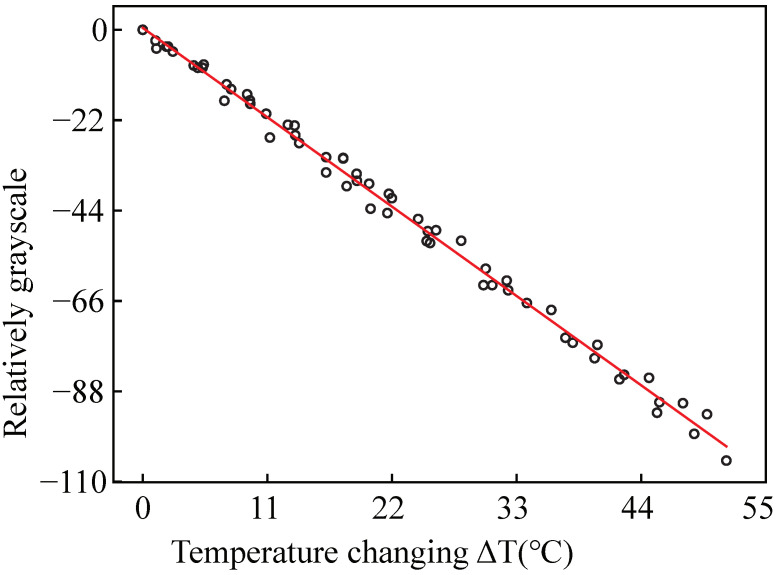
The fitting line of relative grayscale and the temperature change.

The grayscale of the image is proportional to the luminescent intensity [32]. The images of luminescent intensity were imported into MATLAB and translated into intensity images. We selected the luminescent intensity area and took the average grayscale. A line was fitted by the relative average grayscale and the temperature change, as shown in Figure 4. The coefficients of c and d can be obtained from the line: c = 0.27733304 and d = −1.9669431.

### 3.2. Fluid Temperature Measurement

**Figure 5:**
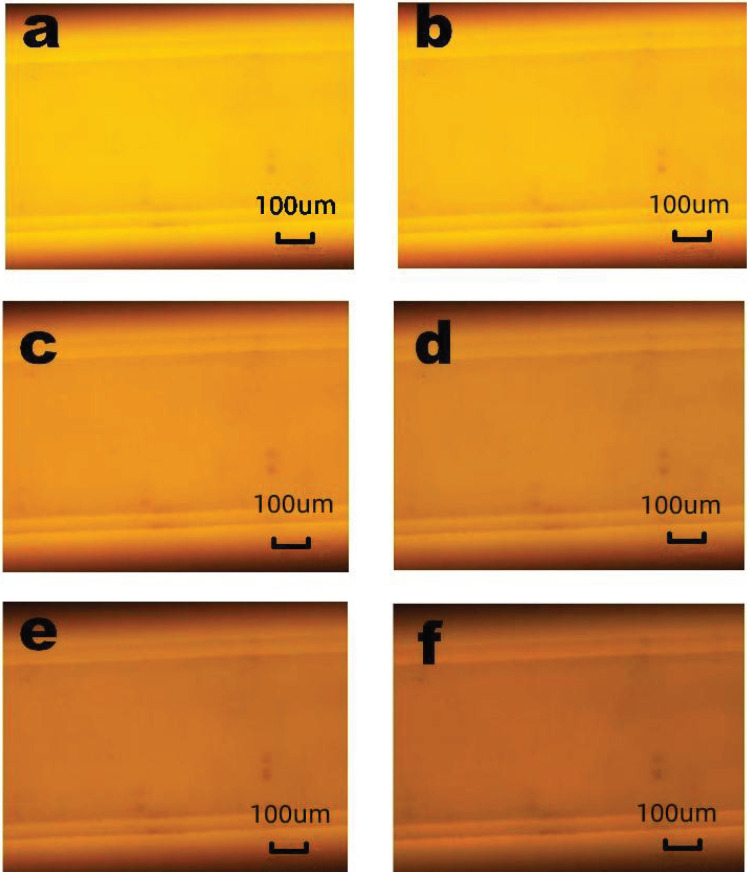
The RhB luminescent intensity pictures with time of (**a**) 0 s, (**b**) 20 s, (**c**) 40 s, (**d**) 60 s, (**e**) 80 s, and (**f**) 100 s when the input voltage was 0.7 V. The bar is 100 μm.

**Figure 6:**
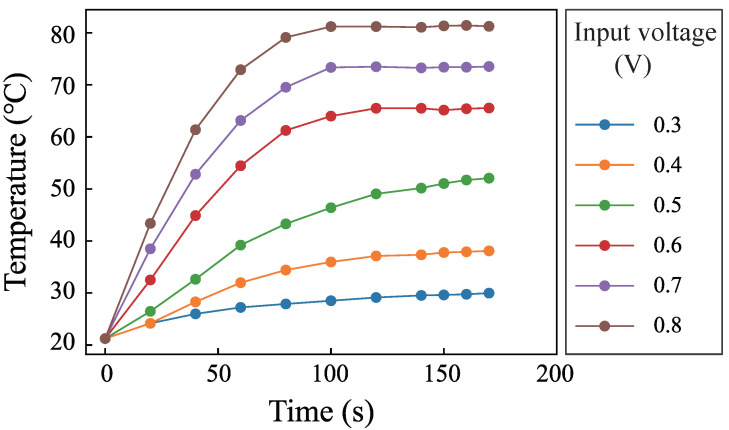
Fluid temperature changing with time when the input voltages are 0.3 V, 0.4 V, 0.5 V, 0.6 V, 0.7 V, and 0.8 V.

The pump pumped the RhB solution into the channel. Then, the pump was closed. SSAWs were generated to heat the fluid. The frequency was 15.59 MHz. The input voltages of the generator were 0.3 V, 0.4 V, 0.5 V, 0.6 V, 0.7 V, and 0.8 V. Figure 5 shows the changing of the luminescent intensity with time when the input voltage was 0.6 V. The images were imported into MATLAB to obtain gray values. The temperature can be obtained by putting the gray value onto the line in Figure 4. The fluid temperature with different times can be used to describe the temperature rise process. Figure 6 shows the temperature rise process when the input voltage were 0.3 V, 0.4 V, 0.5 V, 0.6 V, 0.7 V, and 0.8 V. The results show that SSAWs can heat the fluid to a high temperature when the fluid is still. Many acoustofluidics devices are used in biological and chemical reactions, in which the temperature is an important parameter. It is necessary to integrate the temperature controlling system in the acoustofluidics when the fluid is still.

**Figure 7:**
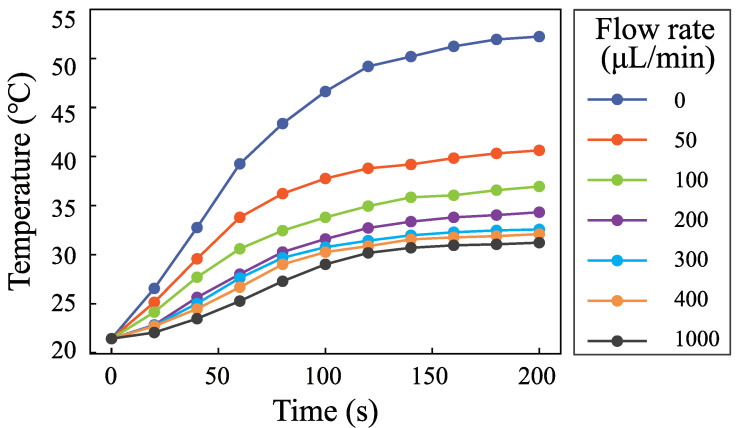
Fluid temperature changing with time when the input voltage was 0.5 V; the flow rates were 0 μL· min${}^{-1}$, 50 μL· min${}^{-1}$, 100 μL· min${}^{-1}$, 200 μL· min${}^{-1}$, 300 μL· min${}^{-1}$, 400 μL· min${}^{-1}$, and 1000 μL· min${}^{-1}$.

**Figure 8:**
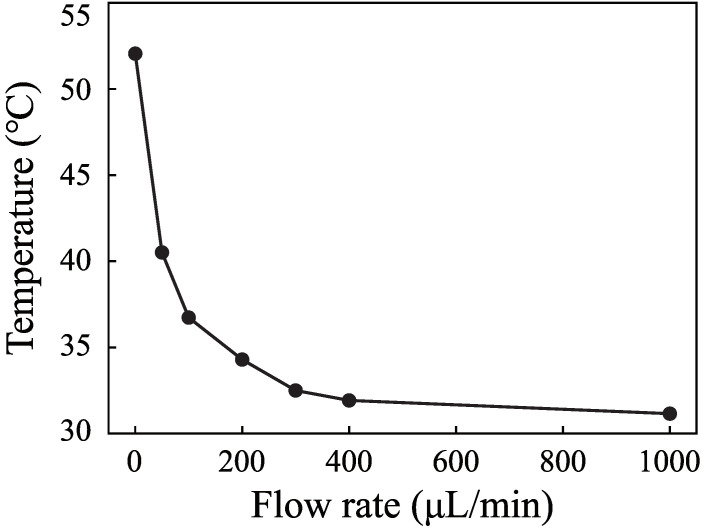
The highest fluid temperature of the different flow rates when the input voltage was 0.5 V.

The flow rate is an important parameter in microfluidics. It is important to measure the temperature with different flow rates. Figure 7 shows the temperature rise process when the input voltage is 0.5 V and the flow rates are 0 μL· min${}^{-1}$, 50 μL· min${}^{-1}$, 100 μL· min${}^{-1}$, 200 μL· min${}^{-1}$, 300 μL· min${}^{-1}$, 400 μL· min${}^{-1}$, and 1000 μL· min${}^{-1}$. Figure 8 shows the change of the highest temperature in Figure 7 with the flow rates. The temperature decreases from 55 ${}^{\circ }$C to 32 ${}^{\circ }$C when the flow rate increases from 0 μL· min${}^{-1}$ to 400 μL· min${}^{-1}$. The temperature decreases rapidly with the increase of the flow rate. The temperature decreases from 32${}^{\circ }$C to 31${}^{\circ }$C when the flow rate increases from 400 μL· min${}^{-1}$ to 1000 μL· min${}^{-1}$. The role of fluid flow in the cooling fluid can be neglected. The temperature is 11 ${}^{\circ }$C higher than the environmental temperature. The results demonstrate that SSAWs can heat the fluid to a high temperature rapidly, which will limit the application of SSAWs in biological and chemical reactions. The flow rate plays an important role in cooling fluid. It is necessary to take into account the role of flow rate in the controlling system.

## 4. Conclusions

In this paper, the fluid temperature in the microchannel heated by SSAWs was measured by the fluorescent signal intensity ratios of Rhodamine B. First, a thermocouple was integrated into the microfluidics to calibrate the relationship between the fluorescent signal intensity ratios of Rhodamine B and the fluid temperature. Then, the fluorescent signal intensity ratios of Rhodamine B were used to measure the fluid temperature heated by the SSAWs. Finally, the fluid temperature with different input voltages and different flow rates was measured by the fluorescent signal intensity ratios of Rhodamine B. The results show that SSAWs can heat the still fluid rapidly to 80${}^{\circ }$C, and the flow rates will influence the temperature of the fluid. The results will be useful for precisely controlling the temperature of acoustofluidics devices.

## Data Availability

The data presented in this study are available on request from the corresponding author.

## References

[1] Zheng T., Wang C., Xu C., Hu Q. (2020). Ultrafast crystallization hollow nanocrystals of the resorcinarene hexamer in microfluidic via standing surface acoustic waves (SSAWs). Mater. Lett..

[2] Xu C., Wang C., Zheng T., Hu Q., Bai C. (2018). Surface acoustic wave (SAW)-induced synthesis of HKUST-1 with different morphologies and sizes. CrystEngComm.

[3] Tengfei Z., Chaohui W., Baogang M., Zhuangde J. (2016). Isolation of sodium chloride crystals induced by standing surface acoustic waves (SSAWs) in a drying droplet. CrystEngComm.

[4] Tayebi M., O’Rorke R., Wong H.C., Low H.Y., Han J., Collins D.J., Ai Y. (2020). Massively multiplexed submicron particle patterning in acoustically driven oscillating nanocavities. Small.

[5] Ma Z., Holle A.W., Melde K., Qiu T., Poeppel K., Kadiri V.M., Fischer P. (2020). Acoustic holographic cell patterning in a biocompatible hydrogel. Adv. Mater..

[6] Dai Nguyen T., Fu Y.Q., Tran V.T., Gautam A., Pudasaini S., Du H. (2020). Acoustofluidic closed-loop control of microparticles and cells using standing surface acoustic waves. Sens. Actuators B Chem..

[7] Connacher W., Zhang N., Huang A., Mei J., Zhang S., Gopesh T., Friend J. (2018). Micro/nano acoustofluidics: Materials, phenomena, design, devices, and applications. Lab Chip.

[8] Baudoin M., Thomas J.L. (2020). Acoustic tweezers for particle and fluid micromanipulation. Annu. Rev. Fluid Mech..

[9] Fu Y.Q., Luo J., Nguyen N.T., Walton A., Flewitt A.J., Zu X.T., Li Y., McHale G., Matthews A., Iborra E. (2017). Advances in piezoelectric thin films for acoustic biosensors, acoustofluidics and lab-on-chip applications. Prog. Mater. Sci..

[10] Skov N.R., Sehgal P., Kirby B.J., Bruus H. (2019). Three-dimensional numerical modeling of surface-acoustic-wave devices: Acoustophoresis of micro-and nanoparticles including streaming. Phys. Rev. Appl..

[11] Zheng T., Wang C., Xu C., Hu Q., Wei S. (2018). Patterning microparticles into a two-dimensional pattern using one column standing surface acoustic waves. Sens. Actuators A Phys..

[12] Tay A.K., Dhar M., Pushkarsky I., Di Carlo D. (2015). Research highlights: Manipulating cells inside and out. Lab Chip.

[13] Zhang P., Bachman H., Ozcelik A., Huang T.J. (2020). Acoustic Microfluidics. Annu. Rev. Anal. Chem..

[14] Shilton R.J., Mattoli V., Travagliati M., Agostini M., Desii A., Beltram F., Cecchini M. (2015). Rapid and controllable digital microfluidic heating by surface acoustic waves. Adv. Funct. Mater..

[15] Kondoh J., Shimizu N., Matsui Y., Sugimoto M., Shiokawa S. (2009). Development of temperature-control system for liquid droplet using surface acoustic wave devices. Sens. Actuators A Phys..

[16] Roux-Marchand T., Beyssen D., Sarry F., Elmazria O. (2015). Rayleigh surface acoustic wave as an efficient heating system for biological reactions: Investigation of microdroplet temperature uniformity. IEEE Trans. Ultrason. Ferroelectr. Freq. Control.

[17] Zhang A.L., Wei Y.Q., Han Q.J. (2012). A microreactor with surface acoustic wave micro-heating system. Ferroelectrics.

[18] Meng L., Deng Z., Niu L., Li F., Yan F., Wu J., Cai F., Zheng H. (2015). A disposable microfluidic device for controlled drug release from thermal-sensitive liposomes by high intensity focused ultrasound. Theranostics.

[19] Kulkarni K., Friend J., Yeo L., Perlmutter P. (2014). An emerging reactor technology for chemical synthesis: Surface acoustic wave-assisted closed-vessel Suzuki coupling reactions. Ultrason. Sonochem..

[20] Xu G., Gunson R.N., Cooper J.M., Reboud J. (2015). Rapid ultrasonic isothermal amplification of DNA with multiplexed melting analysis–applications in the clinical diagnosis of sexually transmitted diseases. Chem. Commun..

[21] Reboud J., Bourquin Y., Wilson R., Pall G.S., Jiwaji M., Pitt A.R., Graham A., Waters A.P., Cooper J.M. (2012). Shaping acoustic fields as a toolset for microfluidic manipulations in diagnostic technologies. Proc. Natl. Acad. Sci. USA.

[22] Ha B.H., Lee K.S., Destgeer G., Park J., Choung J.S., Jung J.H., Shin J.H., Sung H.J. (2015). Acoustothermal heating of polydimethylsiloxane microfluidic system. Sci. Rep..

[23] Park J., Jung J.H., Destgeer G., Ahmed H., Park K., Sung H.J. (2017). Acoustothermal tweezer for droplet sorting in a disposable microfluidic chip. Lab Chip.

[24] Park J., Ha B.H., Destgeer G., Jung J.H., Sung H.J. (2016). Spatiotemporally controllable acoustothermal heating and its application to disposable thermochromic displays. RSC Adv..

[25] Richard C., Fakhfouri A., Colditz M., Striggow F., Kronstein-Wiedemann R., Tonn T., Medina-Sánchez M., Schmidt O.G., Gemming T., Winkler A. (2019). Blood platelet enrichment in mass-producible surface acoustic wave (SAW) driven microfluidic chips. Lab Chip.

[26] Wu M., Huang P.H., Zhang R., Mao Z., Chen C., Kemeny G., Li P., Lee A.V., Gyanchandani R., Armstrong A.J. (2018). Circulating Tumor Cell Phenotyping via High-Throughput Acoustic Separation. Small.

[27] Wang Y., Chen D., Chen X., Li D., Wu C., Xie J. (2019). A surface acoustic wave device for water impurity levels monitoring by measuring signal-to-perturbation ratios. Jpn. J. Appl. Phys..

[28] Zheng T., Wang C., Hu Q., Wei S. (2018). The role of electric field in microfluidic heating induced by standing surface acoustic waves. Appl. Phys. Lett..

[29] Tao X., Dai Nguyen T., Jin H., Tao R., Luo J., Yang X., Torun H., Zhou J., Huang S., Shi L. (2019). 3D patterning/manipulating microparticles and yeast cells using ZnO/Si thin film surface acoustic waves. Sens. Actuators B Chem..

[30] Nefzi A., Carr L., Dalmay C., Pothier A., Leveque P., Arnaud-Cormos D. (2019). Microdosimetry Using Rhodamine B Within Macro-and Microsystems for Radiofrequency Signals Exposures of Biological Samples. IEEE Trans. Microw. Theory Tech..

[31] Behm L.V., Schlenther I., Petrausch M., Jorde F., Godino N., Pfisterer F., Duschl C., Kirschbaum M. (2018). A simple approach for the precise measurement of surface temperature distributions on the microscale under dry and liquid conditions based on thin Rhodamine B films. Sens. Actuators B Chem..

[32] Shah J.J., Gaitan M., Geist J. (2009). Generalized temperature measurement equations for rhodamine B dye solution and its application to microfluidics. Anal. Chem..

[33] Claucherty S., Sakaue H. (2017). An optical-chemical sensor using rhodamine B on anodized-aluminum for surface temperature measurement from 150 to 500 K. Sens. Actuators B Chem..

